# Real-world effect of intermittent calorie-restricted diet on type 2 diabetes remission: a dual-cohort retrospective study

**DOI:** 10.3389/fnut.2025.1648314

**Published:** 2025-09-22

**Authors:** Zhiyong Xiao, Xinhong Yin, Xihu Lai, Xu Zhou, Yewu Zhang, Dongliang Yang, Ruiyu Wu, Huiqing Wang, Jiali Zhou, Xiao Yang, Bin Zhou, Wu Luo, Xuan Chen, Dongbo Liu

**Affiliations:** ^1^Horticulture College, Hunan Agricultural University, Changsha, China; ^2^School of Nursing, University of South China, Hengyang, China; ^3^Xincheng Smart Internet Hospital, Chengdu, China; ^4^Yuelushan Laboratory, Changsha, China; ^5^Chinese Center for Disease Control and Prevention, Beijing, China; ^6^Department of General Education Courses, Cangzhou Medical College, Cangzhou, China; ^7^Hunan Provincial Engineering Research Centre of Medical Nutrition Intervention Technology for Metabolic Diseases, Changsha, China; ^8^Hepatobiliary and Pancreatic Surgery Department, First Hospital of University of South China, Hengyang, China; ^9^Department of Endocrinology, The Central Hospital of Shaoyang, Shaoyang, China

**Keywords:** dual-cohort retrospective study, diet, intermittent calorie-restricted diet, diabetes remission, real-world

## Abstract

**Aims:**

Randomized controlled trials (RCTs) have demonstrated that intermittent calorie-restricted diet (ICR) can lead to diabetes remission. We aimed to assess the diabetes remission with ICR among patients with type 2 diabetes (T2D) in real-world settings.

**Materials and methods:**

We performed a retrospective, dual-cohort study (January 2022–July 2023) using real-world data from Chinese patients with T2D. The ICR cohort consisted of 1,069 patients following an intermittent calorie-restricted diet, while the control cohort consisted of 1,099 patients receiving Dietary Guidelines for Diabetes in China (2017 Edition). The primary outcome was diabetes remission. Secondary outcomes included reductions in antidiabetic medication use and changes in fasting blood glucose (FBG). Subgroup evaluations for the sensitivity analysis were conducted to further assess outcomes. The study employed a combination of univariate and multivariate analyses, including Linear Mixed-Effects Models (LMM), Generalized Linear Mixed-Effects Models (GLMM), and Cox regression with propensity score-weighted Inverse Probability Weighting (IPW), to evaluate relationships between cohort and outcomes.

**Results:**

In real-world settings, the ICR cohort achieved significantly higher remission rates (20% vs. 2%, *p* < 0.001), greater medication reduction (61% vs. 22%, *p* < 0.001). After IPW adjustment, ICR remained superior for remission (OR: 11.02, 95% CI: 8.12–14.96) and reduce medication usage (Estimate: 6.26, 95% CI: 5.61–6.99, *p* < 0.001). Subgroup analyses confirmed consistent benefits across FBG levels and diabetes durations.

**Conclusion:**

This study demonstrated the practical efficacy of ICR in achieving diabetes remission. These findings establish dietary interventions as a powerful and viable strategy for T2D remission.

## 1 Introduction

The rising prevalence of type 2 diabetes (T2D) presents significant risks to individual health and represents a major burden on healthcare systems worldwide (1, 2). Until recently, T2D was largely regarded as a lifelong, progressive condition with inevitable deterioration in glycemic control and increased dependency on pharmacotherapy (3, 4). In response to this growing epidemic, numerous therapeutic strategies have been developed to manage and potentially reverse diabetes. Pharmacological treatments, including insulin and oral glucose-lowering agents, remain the cornerstone of T2D management; however, remission without specific interventions is rare. A U.S. study involving 122,781 adults reported a seven-year cumulative incidence of remission at only 1.60% (5), while data from England covering 2,297,700 individuals with T2D indicated that merely 1.7% met the criteria for diabetes remission (6). Even with intensive diabetes education and enhanced pharmacotherapy, a significant proportion of patients fail to achieve adequate remission.

Randomized controlled trials (RCTs) have explored the potential of intermittent calorie-restricted diet as a viable intervention for diabetes management (7–12). For instance, a group following a diet of 500 to 600 kcal/day for 2 days a week and their usual diet for the remaining 5 days achieved a 19.42% remission rate in obese T2D patients (8). Unlike early results from the NHS Type 2 Diabetes Path to Remission Programme indicate that diabetes remission is achievable outside of research environments, continuous energy restriction has been identified as an acceptable treatment option for certain individuals with T2D (13). However, much of the evidence supporting intermittent calorie-restricted diet (ICR) and continuous energy restriction in diabetes remission comes from highly controlled settings, with limited real-world applicability, evidence on its effectiveness in typical clinical practice remains scarce (14).

One notable RCT conducted in China examined the effects of ICR that participants underwent six cycles of 15-day intermittent calorie restriction, consisting of 5 days of hypocaloric meals (917 kcal/day) followed by 10 days of an unrestricted diet, compared to a control group that received a normal diet. After the 3-month intervention and a subsequent 3-month follow-up, 47.2% of participants in the intervention group achieved diabetes remission, compared to only 2.8% in the control group (9, 11). While these results are promising, they still reflect a controlled research environment with specific inclusion criteria, limiting their applicability to broader, diverse populations.

**Figure 1:**
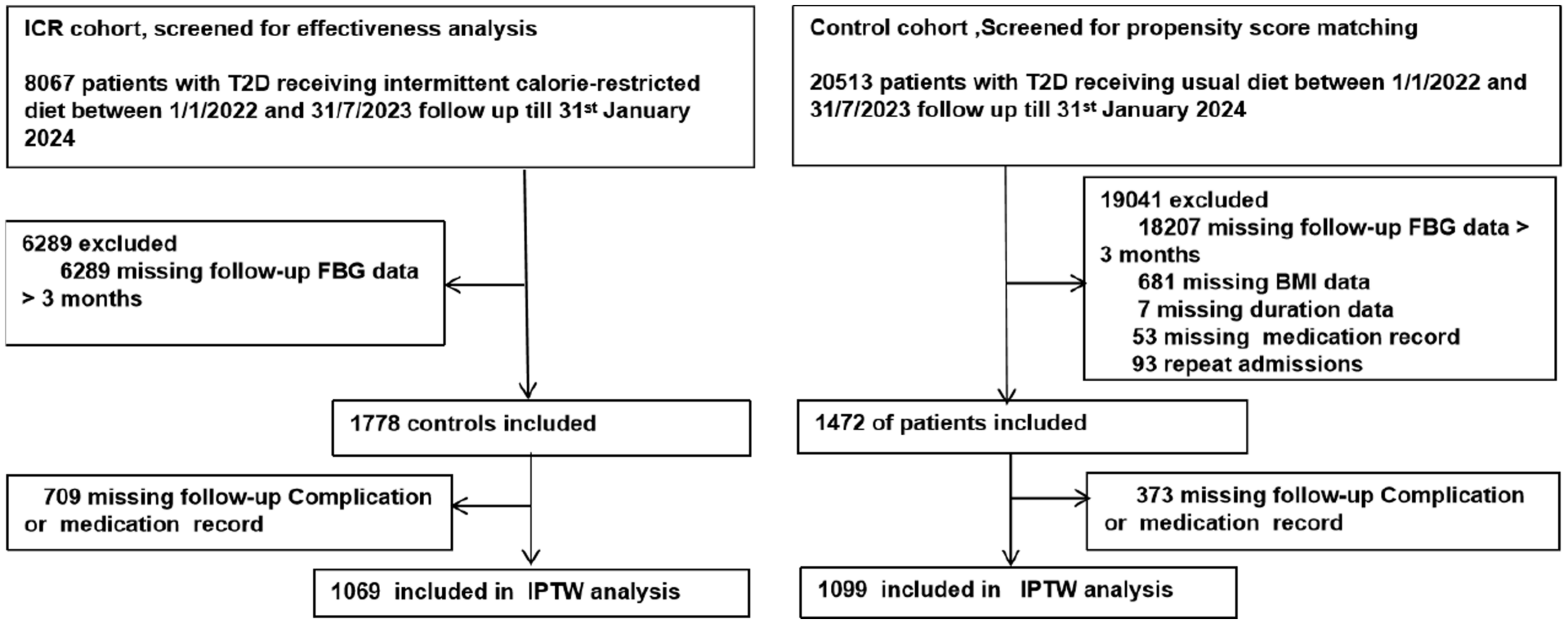
Study profile. This flowchart illustrates the study duration, inclusion and exclusion criteria, and allocation of participants to ICR and control cohorts, along with their respective diabetes remissions and follow-up outcomes.

Building on these promising findings, the Xincheng Intelligent Internet Hospital initiated the ICR Diabetes Remission Program in 2021, extending the intervention to a national scale. This study utilizes data collected over a period of 1 year and 7 months to retrospectively evaluate the real-world impact of intermittent calorie restriction diet on diabetes remission. To provide a comprehensive comparison, a parallel cohort was established by analyzing data from patients receiving conventional diabetes management at the First Affiliated Hospital of the University of South China during the same period. This dual-cohort study aimed to evaluate the real-world effectiveness of dietary intervention in achieving diabetes remission. By leveraging real-world data and a dual-cohort design, we aim to address the gaps in existing literature regarding the practical application of ICR and provide evidence to support its integration into clinical practice as an effective therapeutic strategy (see Figure 1).

## 2 Methods

### 2.1 Study design and data sources

The study period spanned from January 1, 2022, to July 31, 2023, with follow-up concluding on January 31, 2024. For patients with multiple follow-up visits, the longest duration was used for analysis to ensure a comprehensive assessment of intervention effects. Due to the real-world nature of the intervention, randomization was not feasible, inverse Inverse Probability Weighting (IPW) was applied to mitigate selection bias between the ICR and control cohorts. The inclusion criteria for both cohorts were as follows: age between 18 and 75 years, body mass index (BMI) between 18 and 35 kg/m^2^, and a confirmed diagnosis of T2D. Exclusion criteria included participants with less than 3 months of follow-up, as this minimum duration was required to adequately assess diabetes remission status.

The ICR cohort comprised patients enrolled in the Chinese Medical Nutrition Therapy (CMNT) Diabetes Remission Program at Xincheng Intelligent Internet Hospital. The ICR cohort was instructed to consume the provided low-energy CMNT diet (917 kcal/day) as a specific meal replacement for 5 consecutive days, followed by 10 days of regular food intake for 6 cycles (90 days). Dietary composition of CMNT diet was based on wholegrains and homologous substances of medicine and food. The CMNT diet included four ready-to-consume prepared foods: composite nutritional rice, solid beverages, meal replacement biscuits, and fruit and vegetable gruel (detailed in Supplementary eTable 1), followed by 10 days of ad libitum eating, guided by the Dietary Guidelines for Diabetes in China (2017 Edition) as was done for the entire period in the control cohort. Guidelines endorse a fiber-focused, low-glycemic eating pattern: at least 5 g of fiber per serving, carbohydrates providing roughly 50–65% of daily energy, protein 15–20%, and total fat 20–30%, with saturated fat held below 7% of total calories (11). During the fasting days, participants were instructed to start eating breakfast between 06:30 and 08:30, with lunch and dinner scheduled between 11:00–13:00 and 17:00–19:00, respectively. To ensure adherence, health managers trained with the Clinical Standard Protocol supervised participants in the ICR cohort by digital delivery model via videoconferencing. Within the remote management setting, dietary and physical activity adherence data were not captured. Medication management was overseen by physicians, who adjusted or discontinued antidiabetic medications in line with established protocols from previous RCTs, ensuring patient safety and adherence to intervention standards.

The control cohort consisted of outpatient patients from the First Affiliated Hospital of the University of South China, receiving normal diet. The same inclusion and exclusion criteria were applied, resulting in a final cohort of 1,099 patients selected from an initial pool of 20,541. The control cohort received standard diabetes care, which included diet guidance, medication, and regular monitoring as per the local clinical guidelines. This ensured comparability between the cohorts in terms of baseline characteristics and treatment conditions during the follow-up period.

Data collection for both cohorts included demographic and clinical characteristics, such as gender, age, BMI, diabetes duration, glycated hemoglobin A_1c_ (HbA_1c_) levels, fasting blood glucose (FBG), medication use, presence of complications, remission status, and medical costs. The baseline and follow-up clinical assessments were conducted using standardized protocols to ensure consistency. Handling of Missing Data: Missing data accounted for less than 1% of the dataset. For variables where data were sporadically missing, multiple imputation was employed to handle missing values to minimize the impact on statistical power, for continuous variables, medians were used as imputations, while for categorical variables, modes were used. However, for assessing diabetes remission outcomes, we adopted a conservative approach, classifying patients with missing follow-up data as not achieving remission. This approach was chosen to avoid the potential biases introduced by imputing critical outcome data, thereby enhancing the robustness of conclusions related to primary endpoints. By applying the same inclusion and exclusion criteria across both cohorts and rigorously handling missing data, this aimed to enhance comparability, reproducibility, and reliability in evaluating the real-world effectiveness of intermittent fasting compared to conventional diabetes management.

### 2.2 Procedures

A retrospective cohort study design was used as the primary analysis, with subgroup analyses serving as sensitivity checks to validate the robustness of the findings. Univariate analyses were conducted to compare baseline characteristics and outcomes between the dietary intervention and control cohorts. Significant differences in region, age, follow-up duration, FBG, complications, insulin use, and number of medications (*p* < 0.05) were adjusted for in the final models to control for confounding. Generalized Linear Mixed-Effects Models (GLMM) which used to assess the primary outcome of diabetes remission was selected due to its capacity to incorporate both fixed effects, such as treatment group and clinical variables, and random effects, such as region. This approach effectively adjusts for geographic variations in patient populations and healthcare practices. Furthermore, GLMM facilitates the analysis of both continuous outcomes, such as changes in FBG, and binary outcomes, such as remission. Covariates included treatment group, follow-up duration, baseline FBG, complications, insulin use, age, and medication count. IPW was applied to balance baseline covariates between cohorts, enhancing comparability and supporting valid causal inferences regarding the effects of the intervention on outcomes.

To evaluate the consistency and robustness of the intervention effects, subgroup analyses were conducted as part of the sensitivity analysis. These analyses assessed the intervention’s impact across various patient subgroups, categorized as follows: insulin use (insulin users versus non-users), diabetes duration (greater than 5 years versus 5 years or less), BMI (less than 24 kg/m^2^ versus 24 kg/m^2^ or greater), medication use (patients using multiple medications versus those using fewer medications), and FBG at follow-up (less than 7 mmol/L versus 7 mmol/L or greater).

This study adhered to the Strengthening the Reporting of Observational Studies in Epidemiology (STROBE) guidelines. Ethical approval was obtained from the Medical Ethics Committee of the University of South China (Approval No. 2023NHHL090). As the study involved anonymized, retrospective data, patient consent was not required.

### 2.3 Outcomes

The primary outcome was diabetes remission, defined as the discontinuation of glucose-lowering medications for at least 3 months, with HbA_1c_ < 6.5%, based on the 2021 guidelines for diabetes remission (15). Secondary outcomes included: (1) Reduction in diabetes medication use, defined as a decrease in the number of medications between baseline and follow-up; (2) Change in FBG, calculated as the difference between baseline and follow-up levels.

### 2.4 Statistical analysis

In this study, univariate analysis was conducted using the CBCgrps package in R. Continuous variables that adhered to a normal distribution are presented as mean ± standard deviation (Mean ± SD). For continuous variables deviating from normal distribution, the median and interquartile range (M [Q1, Q3]) are provided. Categorical variables are described using frequency (n) and percentage (%). To evaluate differences in baseline characteristics across various groups, a one-way analysis was performed using independent samples t-test, Mann–Whitney U test, and chi-square test (χ^2^ test), as appropriate. To further explore the relationships between independent and dependent variables, multivariate analysis was performed using Linear Mixed-Effects Models (LMM) and GLMM for continuous and binary dependent variables, respectively. These models were developed using the lme4 package in R, which effectively addresses random effects and repeated measures within the data.

GLMM was specifically selected for analyzing diabetes remission due to its ability to account for both fixed effects (e.g., treatment groups, clinical covariates) and random effects (e.g., region). This flexibility is crucial when dealing with real-world data, where variability between study sites and differences in patient populations can introduce significant heterogeneity. By treating region as a random effect, GLMM allows for adjusting the influence of unobserved regional factors without explicitly modeling them as fixed effects. Additionally, GLMM is well-suited for handling both continuous and categorical dependent variables in a unified framework, making it preferable over traditional regression models, which are often unable to accommodate such complexity without losing statistical power. This adaptability is particularly important in evaluating diabetes remission, where the outcome involves both clinical measurements and binary success indicators. To mitigate the impact of baseline imbalances between the intervention and control cohorts, IPW was employed using the PS weight package in R. IPW was chosen to simulate the balance that randomization typically provides in clinical trials, thereby reducing selection bias inherent in observational studies. By employing weights derived from propensity scores estimated through logistic regression models, IPW enhances the accuracy of comparisons between dietary intervention and control groups. Traditional approaches, such as covariate adjustment, may encounter challenges in adequately balancing complex and numerous baseline differences. In contrast, IPW directly models the probability of treatment assignment, thereby achieving effective balance of observed covariates across groups. This ensures that differences in outcomes can be more confidently attributed to the intervention rather than to pre-existing differences between cohorts. Following the balancing of cohorts through IPW, subsequent analyses were performed utilizing Cox regression and linear regression models to evaluate the relationships between the intervention and various clinical outcomes.

Subgroup analyses were performed to evaluate specific population characteristics and their influence on outcomes. The incidence rates for each subgroup were computed, and a propensity score-weighted Cox proportional hazards regression model was used to evaluate the hazard ratios (HR) for diabetes remission and other outcomes within these subgroups. Subgroups were delineated based on baseline characteristics, such as BMI and diabetes duration, to determine how these factors affected the effectiveness of the intervention. Subgroup analysis is essential in identifying potential heterogeneity in treatment effects, allowing the study to understand which patient characteristics may predict greater or lesser response to the dietary intervention. For instance, differences in effectiveness between subgroups with varying BMIs or diabetes durations can indicate that specific patient populations may benefit more from the intervention, thereby informing personalized treatment recommendations. Individual propensity scores were estimated using logistic regression, followed by IPW to adjust for confounding factors in each subgroup. Subsequently, Cox regression analyses were performed for each subgroup, and the weighted hazard ratios, along with their 95% confidence intervals, were reported to quantify the effects of the intervention. These analyses help validate the robustness of the main findings by ensuring that the observed treatment effects are consistent across diverse patient characteristics, supporting the generalizability of the study’s conclusions.

All statistical analyses were conducted using R software (version 4.2.3), with statistical significance set at *α* = 0.05. This comprehensive approach to statistical analysis, leveraging advanced models and weighting techniques, ensures that the findings are both rigorous and applicable in real-world clinical settings.

## 3 Results

The ICR cohort included 1,778 individuals, with 1,069 (60.12%) completing the intervention and follow-up. The cohort had an average diabetes duration of 5 years, 156 (14.59%) used insulin, and the average age was 55 years. Among them, 319 (29.84%) discontinued medications for over 3 months, and 213 (19.92%) achieved HbA_1c_ < 6.5%. The control cohort comprised 1,099 participants, with only 27 (2.46%) achieving HbA_1c_ < 6.5% and 20 (1.82%) maintaining medication cessation for over 3 months (Table 1). Baseline characteristics showed significant differences in age, follow-up duration, FBG, complications, insulin use, and antidiabetic medication use (Supplementary eTable 2).

**Table 1:** Baseline characteristics of ICR and control cohorts.

Variables	Total (*n* = 2,168)	ICR (*n* = 1,069)	Control (*n* = 1,099)	*p*-value
Sex				0.093
Male	1,358 (63)	689 (64)	669 (61)	
Female	810 (37)	380 (36)	430 (39)	
Follow-up duration (days)	171.94 (124.28, 242)	152.21 (119.89, 200.03)	200 (135, 304.5)	< 0.001
FBG at baseline (mmol/L)	8.6 (7.1, 11.4)	7.9 (6.6, 9.7)	9.12 (7.98, 13.61)	< 0.001
Complications at baseline				< 0.001
0	1788 (82)	1,069 (100)	719 (65)	
1	361 (17)	0 (0)	361 (33)	
2	19 (1)	0 (0)	19 (2)	
Insulin use at baseline				< 0.001
Untreated	1,561 (72)	913 (85)	648 (59)	
Treated	607 (28)	156 (15)	451 (41)	
Age (years)	58 (51, 64)	55 (50, 60)	60 (54, 68)	< 0.001
BMI (kg/m^2^)	23.64 (21.66, 25.59)	23.53 (21.8, 25.35)	23.81 (21.48, 25.93)	0.486
Antidiabetic drugs at baseline				< 0.001
0	313 (14)	245 (23)	68 (6)	
1	906 (42)	367 (34)	539 (49)	
2	680 (31)	335 (31)	345 (31)	
3	217 (10)	98 (9)	119 (11)	
4	52 (2)	24 (2)	28 (3)	
Duration of diabetes (years)	5 (2, 10)	5 (2, 10)	6 (1, 10)	0.112

This table presents baseline demographic and clinical characteristics of ICR and control cohorts, including age, FBG, BMI, duration of diabetes, insulin use, and antidiabetic medication use. Statistically significant differences between cohorts are indicated by *p* < 0.05.

Univariate analyses (Table 2) indicated similar FBG reductions in both cohorts (median decrease of 1.4 mmol/L). However, the ICR cohort had significantly higher remission rates (20% vs. 2%, *p* < 0.001), greater medication reduction (62% vs. 22%, *p* < 0.001).

**Table 2:** Univariate analysis of outcomes for ICR and control cohorts.

Variables	Total (*n* = 2,168)	ICR (*n* = 1,069)	Control (*n* = 1,099)	*p*-value
Diabetes remission				< 0.001
No remission	1935 (89)	856 (80)	1,079 (98)	
Remission	233 (11)	213 (20)	20 (2)	
FBG change	−1.4 (−3.2, 0.1)	−1.4 (−2.9, −0.1)	−1.4 (−3.72, 0.86)	0.346
Drugs reduction				< 0.001
Reduce	888 (41)	651 (61)	237 (22)	
No reduction	1,280 (59)	418 (39)	862 (78)	

Univariate analysis comparing FBG changes, and drugs reduction between ICR and control cohorts. *p*-values below 0.05 indicate statistically significant differences between the cohorts.

Multivariate analysis (Supplementary eTable 3) confirmed superior outcomes in the ICR cohort. The odds of remission were 4.2 times higher (OR: 4.2, 95% CI: 2.39–7.40, *p* < 0.001), and the likelihood of medication reduction was 18.74 times greater (OR: 18.74, 95% CI: 7.88–44.58, *p* < 0.001). Higher baseline FBG was associated with decreased remission probability (OR: 0.83, 95% CI: 0.77–0.89, *p* < 0.001), and baseline insulin use was linked to lower remission odds (OR: 0.20, 95% CI: 0.08–0.52, *p* = 0.001). Age negatively correlated with remission (OR: 0.98, 95% CI: 0.95–1.00, *p* = 0.035).

After applying IPW (Supplementary eFigure 1), covariate imbalances were substantially reduced. No significant difference in FBG change was observed (Estimate: -0.07, 95% CI: −0.46 to 0.32, *p* = 0.740). The ICR cohort remained significantly more likely to achieve remission (Estimate: 11.02, 95% CI: 8.12–14.96, *p* < 0.001) and reduce medication usage (Estimate: 6.26, 95% CI: 5.61–6.99, *p* < 0.001) (Table 3).

**Table 3:** Outcomes after IPW.

Variables	Estimate (95% CI)	Std. error	*p*-value
Diabetes remission	11.02 (8.12–14.96)	1.72	< 0.001
FBG change	−0.07 (−0.46 to 0.32)	0.20	0.740
Drugs reduction	6.26 (5.61–6.99)	0.35	< 0.001

This table presents diabetes remission rates, reduction in medication use, and changes in FBG after adjustment for confounders using IPW. Estimates and 95% CIs are provided, with statistical significance indicated by *p* < 0.05.

Subgroup analysis (Table 4) presents a detailed subgroup analysis of outcomes following IPW for both the ICR and control cohorts. The table is organized into three main sections: diabetes remission, FBG change and drug reduction. Across all stratifications, the incidence rate of diabetes remission was significantly higher in the ICR group than in the control group, regardless of baseline BMI, duration of diabetes, antidiabetic drugs, FBG follow-up and insulin treatment. The largest relative effect on remission was observed among participants with a diabetes duration ≥5 years, where the remission incidence rate in the ICR group was 25.48 times that of the control group (95% CI: 6.28–103.35, *p* = 0.001), suggesting that even individuals with long-standing diabetes may benefit substantially from the intervention. There was no statistically significant interaction across most subgroups (all *p*-interaction > 0.05), indicating a consistent treatment effect. Regarding fasting blood glucose (FBG) changes, the ICR group exhibited significantly greater reductions in FBG across nearly all subgroups. Notably, a significant interaction effect was observed in the subgroup stratified by diabetes duration and FBG follow-up frequency (*p*-interaction < 0.001), implying that the degree of FBG improvement may vary by disease duration or follow-up intensity. In terms of medication reduction, the ICR group showed markedly greater reductions compared to the control group in all evaluable subgroups, with consistent effect sizes and no significant interactions. These findings demonstrate the broad and robust effectiveness of the intervention, regardless of baseline clinical characteristics.

**Table 4:** Subgroup analysis of outcomes After IPW for ICR and control cohorts.

Outcome	ICR	Control	ICR versus control	*p*-value	*p*-interaction
	Crude incidence rate (95% CI)	Crude incidence rate (95% CI)	Estimate (95% CI)		
Diabetes remission					
BMI ≥ 24	0.54 (0.44–0.64)	0.03 (0.01–0.05)	6.40 (4.39–9.32)	< 0.001	0.398
BMI < 24	0.36 (0.29–0.43)	0.02 (0.01–0.04)	9.75 (5.80–16.38)	< 0.001	
Complications of diabetes at Baseline = 0	0.43 (0.38–0.49)	0.03 (0.02–0.05)	6.32 (4.61–8.67)	< 0.001	
Duration of diabetes ≥ 5	0.16 (0.16–0.21)	0 (0–0.01)	25.48 (6.28–103.35)	0.001	0.235
Duration of diabetes < 5	0.65 (0.56–0.75)	0.05 (0.03–0.08)	7.26 (5.31–9.93)	< 0.001	
With antidiabetic drugs	0.17 (0.13–0.21)	0.27 (0.11–0.43)	5.64 (3.57–8.92)	< 0.001	0.906
Without antidiabetic drugs	1.53 (1.28–1.78)	0.013 (0.01–0.02)	4.99 (3.32–7.50)	< 0.001	
FBG follow-up ≥ 7	0.12 (0.07–0.17)	0.01 (0.003–0.02)	4.97 (2.74–9.04)	< 0.001	0.86
FBG follow-up < 7	0.62 (0.53–0.70)	0.07 (0.03–0.11)	5.04 (3.53–7.19)	< 0.001	
With insulin treatment	0.05 (0.001–0.09)	0 (0–0.01)	7.57 (1.60–35.61)	0.08	0.948
Without insulin treatment	0.51 (0.44–0.58)	0.05 (0.03–0.07)	6.43 (4.73–8.75)	< 0.001	
FBG change					
BMI ≥ 24	−1.787 (−2.162 to −1.411)	−1.596 (−2.197 to −0.996)	−1.246 (−1.834 to −0.658)	< 0.001	0.246
BMI < 24	−1.616 (−1.936 to −1.296)	−1.962 (−2.621 to −1.303)	−1.723 (−2.268 to −1.178)	< 0.001	
Complications of diabetes at Baseline = 0	−1.687 (−1.930 to −1.444)	−1.282 (−2.075 to −0.489)	−1.635 (−2.045 to −1.224)	< 0.001	
Duration of diabetes ≥ 5	−1.790 (−2.156 to −1.425)	−0.470 (−1.109 to 0.169)	−3.019 (−3.626 to −2.411)	< 0.001	< 0.001
Duration of diabetes < 5	−1.604 (−1.93 to −1.278)	−3.166 (−3.759 to −2.572)	−0.410 (−0.927 to 0.108)	0.121	
With antidiabetic drugs	−1.545 (−1.820 to −1.270)	−1.716 (−2.180 to −1.252)	−1.530 (−1.983 to −1.076)	< 0.001	0.671
Without antidiabetic drugs	−2.290 (−2.79 to −1.790)	−2.956 (−4.589 to −1.323)	−1.280 (−2.071 to −0.490)	0.002	
FBG follow-up ≥ 7	−0.953 (−1.418 to −0.488)	−0.461 (−0.961 to 0.039)	−3.050 (−3.680 to −2.421)	< 0.001	< 0.001
FBG follow-up < 7	−2.112 (−2.375 to −1.848)	−5.433 (−6.184 to −4.682)	1.395 (0.946 to 1.845)	< 0.001	
With insulin treatment	−1.138 (−1.745 to −0.532)	−0.871 (−1.645 to −0.098)	−1.091 (−1.99 to −0.190)	0.018	0.319
Without insulin treatment	−1.796 (−2.061 to −1.532)	−2.430 (−2.956 to −1.902)	−1.580 (−2.024 to −1.136)	< 0.001	
Drugs reduction					
BMI ≥ 24	1.38 (1.22–1.54)	0.31 (0.25–0.37)	3.27 (2.80–3.82)	< 0.001	0.546
BMI < 24	1.29 (1.16–1.42)	0.36 (0.30–0.42)	2.78 (2.41–3.22)	< 0.001	
Complications of diabetes at Baseline = 0	1.33 (1.22–1.43)	0.32 (0.27–0.38)	2.78 (2.49–3.12)	< 0.001	
Duration of diabetes ≥ 5	1.39 (1.23–1.54)	0.38 (0.31–0.44)	3.01 (2.58–3.50)	< 0.001	0.99
Duration of diabetes < 5	1.28 (1.14–1.41)	0.29 (0.24–0.35)	3.01 (2.60–3.49)	< 0.001	
With antidiabetic drugs	1.63 (1.51–1.76)	0.36 (0.31–0.40)	3.26 (2.93–3.62)	< 0.001	NA
Without antidiabetic drugs	NA	NA	NA	NA	
FBG follow-up ≥ 7	1.23 (1.07–1.39)	0.31 (0.27–0.36)	2.94 (2.54–3.41)	< 0.001	0.686
FBG follow-up < 7	1.38 (1.25–1.51)	0.39 (0.30–0.48)	2.61 (2.22–3.06)	< 0.001	
With insulin treatment	1.69 (1.41–1.98)	0.39 (0.32–0.47)	3.52 (2.91–4.26)	< 0.001	0.449
Without insulin treatment	1.25 (1.14–1.36)	0.29 (0.24–0.35)	2.88 (2.53–3.27)	< 0.001	

Subgroup analyses of clinical outcomes after IPW in the ICR and control cohorts. The table presents crude incidence rates and adjusted estimates with 95% CI for diabetes remission, FBG changes, and medication reduction across subgroups stratified by BMI, duration of diabetes, baseline complications, antidiabetic drug use, insulin treatment, and FBG follow-up levels. p-interaction indicates heterogeneity in treatment effects between subgroups. Data are presented as incidence rates (95% CI) or OR (95% CI), with statistically significant results (*p* < 0.05) highlighted.

## 4 Discussion

The findings from this study provide robust real-world evidence that intermittent energy restriction outperforms usual diet in achieving diabetes remission, reducing medication dependency. This study is the first to demonstrate T2D remission can be achieved through large-scale implementation of ICR outside of a research setting.

The real-world benefits of dietary interventions can be difficult to capture within the constrained timeframes of RCTs due to the prolonged nature of diabetes development and progression (16). Real-world studies are crucial to understanding the broader impacts of interventions on diabetes management (17). The five-year follow-up of the DiRECT trial (18) showed fewer clinical events in patients who successfully achieved remission. The Da Qing trial, which followed a lifestyle intervention which prediabetic individuals received guidance on reducing caloric intake over 30 years, demonstrated a significant reduction in diabetes incidence (19). Similarly, the Diabetes Prevention Program (DPP) demonstrated that intensive lifestyle interventions, including dietary changes, effectively prevented diabetes onset in high-risk individuals (20, 21). Unlike continuous interventions, ICR simplifies dietary management by eliminating daily calorie counting and promoting natural foods over meal replacements, enhancing patient adherence (11, 22). This structured, professionally supported approach yields sustained improvements in well-being and facilitates diabetes remission (11, 22). Notably, studies report sustained diabetes remission after just 3 months of ICR intervention, potentially without ongoing support (11). While real-world ICR completion rates (60.12%) are lower than in controlled trials (88.9%) (11), they exceed those of continuous low-energy diets (13). Patient well-being and engagement are vital for successfully implementing dietary strategies targeting diabetes remission in real-world settings (23). This collaborative approach is essential for the effective and sustainable integration of the intervention into clinical practice, ultimately leading to enhanced health outcomes.

Comparing remission through pharmacotherapy versus dietary intervention, it can be contended that while medication can maintain FBG below 7 mmol/L achieving the same control of glycaemia which is widely regarded as an optimal control target for patients with diabetes (24, 25), this represents disease management rather than true independence. Though achieved similar glycemic control target, ICR offers superior advantages in achieving T2D remission (15). In ICR cohort, 68% of patients were maintained follow-up FBG levels below 7 mmol/L, compared to 44% in the control cohort. Nevertheless, it remains unclear whether achieving comparable glycemic improvement through polypharmacy provides equivalent symptomatic benefits as dietary-induced. While emerging antidiabetic agents like GLP-1 receptor agonists and SGLT2 inhibitors promote symptom control (26, 27), their high cost creates significant barriers to access. In resource-limited settings, ICR represents a clinically effective and economically viable alternative for managing diabetes.

The diabetes remission rate in ICR cohort was lower compared to results from controlled trials, which can be attributed to several real-world challenges. First, the observed reduction in diabetes remission rates within the real-world ICR cohort, compared to controlled trial settings, may be substantially attributable to methodological disparities in adherence monitoring. In the present real-world implementation, health managers delivered protocol-driven supervision exclusively via videoconferencing—a pragmatic approach reflecting clinical practice constraints. Crucially, this remote management model did not incorporate objective capture of dietary or physical activity adherence data, representing a key divergence from RCT methodology where participants submitted private diet diaries to investigators for granular compliance tracking (11). These operational differences contextualize the remission rate disparity and align with the lower completion rates observed in real-world ICR (60.12%) versus RCTs (88.9%). Notably, however, real-world adherence remained superior to continuous low-energy diets, suggesting ICR retains practical advantages despite monitoring limitations. Second, of the 729 patients in the intervention cohort who achieved optimal FBG levels, we were only able to obtain data on over 3 months of medication use and HbA_1c_ levels for 319 patients. To avoid biases associated with statistical imputation or IPW, we did not apply these methods for this missing data. Instead, a conservative approach was employed by classifying patients with missing data as not achieving remission. This may underestimate the true effect but enhances the robustness of our conclusions by minimizing data manipulation biases. This data gap underscores a limitation in real-world studies, where incomplete data can hinder accurate assessments of outcomes. Third, the older baseline age of participants and population heterogeneity impacted remission rates. Unlike RCTs, which tend to include younger and more homogeneous populations, real-world studies must contend with a wide range of patient ages, comorbidities, and varying adherence levels. This heterogeneity makes achieving the high remission rates seen in controlled trials more challenging.

A major strength of this study is the large sample size and inclusion of a control cohort, enabling direct comparison between dietary intervention and conventional diabetes management in a real-world clinical setting. The use of IPW helped mitigate potential selection bias associated with the retrospective study design. Employing both multiple imputation for some variables and a conservative approach for missing remission outcomes, the sensitivity analysis demonstrated that the overall conclusions remained consistent, indicating robustness of the intervention effects. However, the study had several limitations. First, the absence of weight data limits the ability to assess the role of weight loss in diabetes remission, a well-known factor for achieving and sustaining remission (13, 28). Future studies should include weight data to better understand the contribution of weight loss to the success of intermittent fasting. Second, this study was limited by the absence of long-term follow-up data. While significant improvements were observed in the short term, diabetes remission is susceptible to relapse, and without long-term data, the sustainability of remission remains uncertain. The Diabetes Prevention Program Outcomes Study (DPPOS) (19, 29) demonstrated that remission achieved through lifestyle modification, but remission often fluctuates without sustained intervention. Future research should therefore include extended follow-up periods to evaluate the durability of dietary intervention outcomes. In particular, long-term studies should track key clinical endpoints such as sustained glycemic control (e.g., HbA_1c_ < 6.5% without glucose-lowering medication), incidence of cardiovascular events, and changes in quality-of-life indicators. These data will be critical to fully assess the long-term efficacy, safety, and patient-centered benefits of intermittent fasting in the management and potential reversal of type 2 diabetes.

## 5 Conclusion

The ICR is a highly effective dietary intervention for achieving diabetes remission in real-world settings. These findings establish dietary interventions as a powerful and viable strategy for T2D remission, positioning them as an essential component of public health and diabetes care services. Future research must focus on enhancing adherence, ensuring the long-term sustainability of dietary interventions, and integrating it effectively with public health system to achieve the best diabetes management.

## Data Availability

The outcome data and follow-up records were extracted from hospital database. Restrictions apply to the availability of these data, which were used under licence for this study. Requests to access these datasets should be directed to ZX, xiaozy@stu.hunau.edu.cn.
