# Supplementary material for: Real-world effect of intermittent calorie-restricted diet on type 2 diabetes remission: a dual-cohort retrospective study
Source: Front Nutr. 2025 Sep 22;12:1648314. doi: 10.3389/fnut.2025.1648314 (PMC12497798; doi:10.3389/fnut.2025.1648314)
Supplement: Supplementary file 1 [file Data_Sheet_1.PDF]

Supplementary Content

eFigure 1. Standardized Mean Differences (SMD) of Covariates Before and After IPW Adjustment.

eTable 1. Composition of the Human CMNT Diet.

eTable 1-1. Caloric Content of the Human CMNT Diet.

eTable 1-2. Ingredients of the Human CMNT Diet.

eTable 2. Baseline Medication Use in ICR Cohort Compared to Control Cohort.

eTable 3. Multivariate Analysis of Outcome Indicators.

eFigure 1. Standardized Mean Differences (SMD) of Covariates Before and After IPW Adjustment.

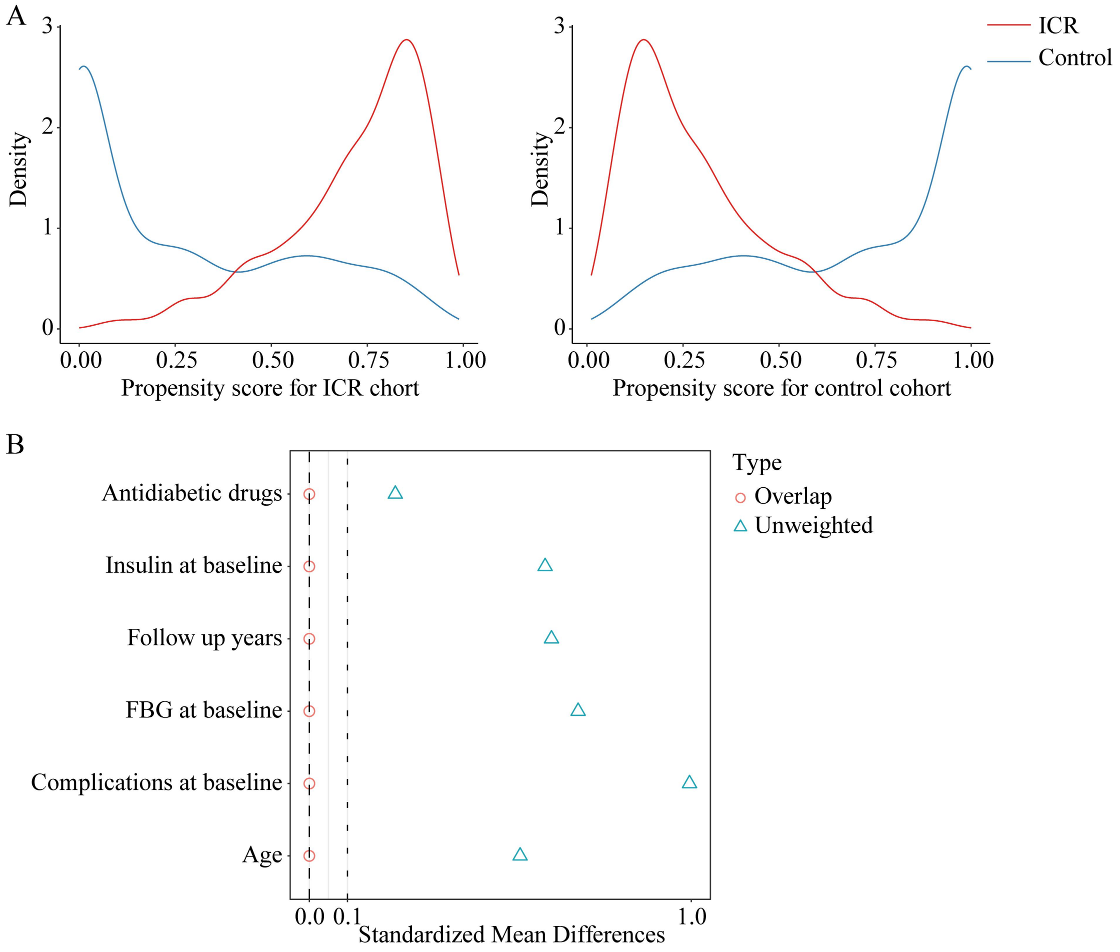

(A) Density plots of propensity scores for the intervention and control cohorts before (left) and after (right) matching. The red solid line represents the ICR cohort, while the blue dashed line indicates the control cohort. Propensity score matching enhanced the alignment of score distribution between the two groups. (B) SMD for covariates before (△) and after (○) propensity score matching. Vertical dashed lines indicate the threshold of 0.1. Post-matching SMD values are closer to zero, demonstrating that matching effectively reduced covariate imbalances and improved comparability between cohorts. Covariates included follow-up duration, baseline FBG, baseline complications, insulin use, age, and medication use.

eTable 1. Composition of the Human CMNT Diet.

eTable 1-1. Caloric Content of the Human CMNT Diet.

**Calorie information**	**Solids beverages**	**Fruit and vegetable gruel**	**Composite nutritional rice**	**Meal replacement biscuit**
Energy density (kcal/100 g)	576.24	533.22	358.75	489.96
Protein (g/100 g)	7.20	3.40	10.50	7.10
Protein %	5.28	2.52	11.86	5.97
Fat (g/100 g)	50.00	30.80	1.80	18.20
Fat %	84.09	52.39	4.66	35.13
Carbohydrates (g/100 g)	14.50	60.80	73.90	70.00
Carbohydrates %	10.63	45.09	83.48	58.90
Fiber (g/100 g)	23.90	-	-	8.20
Sodium (mg/100 g)	63.00	95.00	41.20	264.00

The 5-day CMNT diet (917 kcal/day) comprised four standardized components with the shown nutritional composition per 100g.

eTable 1-2. Ingredients of the Human CMNT Diet.

Diet item	Daily intake	Ingredients
**Breakfast**		
Fruit and vegetable gruel	50 g	Fresh pumpkins, pumpkin seed kernel oil, maltodextrins, isomalto-oligosaccharide, casein, resistant dextrin, sodium ascorbate, potassium citrate, mono- and diglycerides of fatty acids esters, vitamin E, tea polyphenols, and silicon dioxide.
**Lunch**		
Solids beverages	25 g	Pumpkin seed kernel oil, isomalto-oligosaccharide, casein, resistant dextrin, sodium ascorbate, potassium citrate, mono- and diglycerides of fatty acids esters, vitamin E, tea polyphenols, and silicon dioxide.
Composite nutritional rice	60 g	Homologous medicine and food substance: *Fructus lycii*, *Ganoderma lucidum*, Folium Mori, *Poria cocos*, *Dioscorea opposita* Thunb. (Chinese yam), Radix Puerariae, *Cordyceps militaris*, and *Momordica grosvenori*. Wholegrains and others: Rice, millet, corn, buckwheat, quinoa, oat, spinach powders, lily root flour, cucumber powders, mushroom powder, wheat dietary fiber, bitter melon, pumpkins, potato, purple potato, sweet potato, mung bean, konjac flour, inulin, and edible refined salt.
**Dinner**		
Solids beverages	25 g	Pumpkin seed kernel oil, isomalto-oligosaccharide, casein, resistant dextrin, sodium ascorbate, potassium citrate, mono- and diglycerides of fatty acids esters, vitamin E, tea polyphenols, and silicon dioxide.
Meal replacement biscuit	30 g	Medicine food homologous plants, wholegrains, and others: *Dioscorea opposita* Thunb. (Chinese yam), wheat flour, MAIKERENJIA, mix powder (quinoa, white kidney, wheat germ, azuki bean, black beans, yellow beans, liriopes radix, glutinous rice, black rice, maize, round bract *Plantago ovata* husk power, oat, buckwheat, hawthorn, roselle, millet, brown rice, Chinese jujube, Chinese wolfberry, pecan nuts, chia seed, black sesame, white sesame, shiitake mushroom, *Laminaria hyperborea*, and coffee), edible vegetable oils, potato protein, wheat dietary fiber powder, resistant dextrin, maltodextrin, and L-arabinose.

The table details the daily intake and ingredients of the 5-day CMNT diet (917 kcal/day), administered as pre-portioned meals.

eTable 2. Baseline Medication Use in ICR Cohort Compared to Control Cohort.

**Drugs**	**Total number of participants ICR cohort**	**%**	**Total number of participants Control cohort**	**%**
Overall	1,069	100%	1,099	100%
Taking metformin	597	55.85%	408	37.12%
Taking SGLT2 inhibitors	97	9.07%	136	12.37%
Taking DPP-4 inhibitors	47	4.40%	44	4.00%
Taking GLP-1 analogs	5	0.47%	8	0.73%
Taking sulfonylureas	228	21.33%	180	16.38%
Taking pioglitazone	29	2.71%	14	1.27%
Taking meglitinides	32	2.99%	20	1.82%
Taking acarbose	156	14.59%	123	11.19%
Taking insulin	156	14.59%	451	41.04%

This table presents the types of antidiabetic medications used by participants in both cohorts at baseline, highlighting significant differences in drug use between the groups.

eTable 3. Multivariate Analysis of Outcome Indicators.

Predictors	Diabetes remission OR (95% CI)	Diabetes remission *p*-value	FBG change OR (95% CI)	FBG change *p*-value	Drugs reduction Estimates (95% CI)	Drugs reduction *p*-value
ICR	4.2 (2.39–7.40)	< 0.001	4.2 (2.39–7.40)	< 0.001	-1.45 (-1.84 to -1.05)	< 0.001
Follow-up years	0.99 (0.99–0.99)	< 0.001	0.99 (0.99–0.99)	< 0.001	0.00085 (-0.0006 to 0.0023)	0.262
FBG at baseline	0.83 (0.77–0.89)	< 0.001	0.83 (0.77–0.89)	< 0.001	-0.7 (-0.74 to -0.66)	< 0.001
Complications at baseline	1.18 (0.44–3.11)	0.743	1.18 (0.44–3.11)	0.743	0.25 (-0.18 to 0.68)	0.253
Insulin use at baseline	0.2 (0.08–0.52)	0.001	0.2 (0.08–0.52)	0.001	1.18 (0.81–1.56)	< 0.001
Age	0.98 (0.95–1.0)	0.035	0.98 (0.95–1.0)	0.035	0.02 (0.00–0.04)	0.041
Antidiabetic drugs at baseline	0.27 (0.22–0.35)	< 0.001	0.27 (0.22–0.35)	< 0.001	0.003 (-0.17 to 0.17)	0.969

Multivariate analysis of diabetes remission, drug reduction, and FBG changes between the ICR and control cohorts, adjusted for age, baseline FBG, and insulin use. Odds ratios (OR) and 95% confidence intervals (CI) are presented. Significant predictors are indicated with $p < 0.05$. Covariates included treatment group, follow-up duration, baseline FBG, baseline complications, insulin use, age, and medication.
